# Tendon morphological changes after a prolonged ski race can be detected by ultrasound echo intensity

**DOI:** 10.1186/s13047-020-00398-9

**Published:** 2020-06-10

**Authors:** Alessandro Schneebeli, Lorenzo Visconti, Corrado Cescon, Ron Clijsen, Guido Giardini, Maria Elisabetta Arizzio, Marco Barbero

**Affiliations:** 1 Rehabilitation Research Laboratory 2rLab, Department of Business Economics, Health and Social Care, University of Applied Sciences and Arts of Southern Switzerland, Manno/Landquart, Switzerland; 2 Studi fisioterapici di Montagna, Aosta, Italy; 3 International university of applied sciences THIM, Thim van der Laan AG, Landquart, Switzerland; 4 Faculty of Physical Education and Physical Therapy, Vrije Universiteit Brussel, Brussels, Belgium; 5 SC Neurologia Centro Medicina di Montagna, Ospedale U.Parini, Aosta, Italy; 6 FCA group, Torino, Italy

**Keywords:** Achilles tendon, Patellar tendon, Gray scale analysis

## Abstract

**Background:**

Ultrasound imaging techniques have been used to assess the characteristics of skeletal muscles and tendons. Such techniques (gray scale analysis) allow qualitative evaluation and have been used recently to assess the internal structure of muscles and tendons by computer-aided gray scale analysis. We hypothesized that changes in the internal structure of the Achilles and patellar tendons after a ski mountaineering race competition could be detected with ultrasound.

**Methods:**

Twenty athletes were recruited during the 19th Millet Tour du Rutor extreme, a three-day ski mountaineering competition. Ultrasound measurements of the Achilles and patellar tendons were carried out before the first race and immediately after each of the three competition days. Tendon thickness, cross-sectional area (CSA), and ultrasound gray scale analysis were calculated.

**Results:**

Significant differences (*p* < 0.05) were observed between the pre- and post-race measurements for the Achilles tendon thickness and CSA, while no significant differences were noted for the patellar tendon thickness and CSA. However, gray scale analysis of both the Achilles and patellar tendons showed significantly higher post-race values, than the pre-race values (*p* < 0.05).

**Conclusions:**

Achilles and patellar tendons of healthy athletes are highly responsive to an acute increase in mechanical load. Those changes can be detected from classical (thickness and CSA) and innovative (gray scale) ultrasound-based parameters.

**Trial registration:**

This study was approved by the Azienda USL Valle d’Aosta Ethics Committee (protocol no. 23/03/2018.0026243.I).

## Background

In the human musculoskeletal system, tendons have various functions; they transmit forces from muscles to bones, absorb shock, are able to store energy and release and some appear to be positional tendons [1]. Depending on the organization of collagen fibers (orientation, number, and size), tendons can sustain different types of forces [1]. The connective tissue of tendons is not an inert structure, but has considerable capacity to react to external demands by means of dynamic protein turnover [2]. Those adaptations are often due to an increment of mechanical loading applied to the structure that leads to the increased expression of several growth factors [3] and the synthesis of new collagen proteins [2].

The high adaptability of tendon structure to different types of loading and intensity, as well as the morphological and mechanical changes following prolonged training are well known. In comparison to untrained controls, the Achilles tendon (AT) and its cross-sectional area (CSA) in trained endurance and jumping athletes were observed to be larger [4, 5] Moreover, Kongsgaard et al. [6] reported an increase in the CSA of the patellar tendon (PT) after 12 weeks of heavy resistance training. Similarly, Seynnes et al. [7] reported hypertrophy of the PT following short-term resistance training.

Although different studies have reported changes in the morphological properties of tendons, a recent meta-analysis [8] suggests that the adaptive response to mechanical loading could be related to both material properties (i.e., stiffness and Young’s modulus) and morphological characteristics. Indeed, hypertrophy of tendon tissue has been shown to be a subsequent effect of longer periods of loading [9]. The acute effects of training and exercise on morphological properties of the tendon are still unknown. However, some authors postulate that specific morphological changes in the AT and PT are exercise and duration dependent [10]. A reduction in the tendon diameter has been described mostly during repetitive tasks, like eccentric exercises [11], heel raises [12] or squatting exercises [13].

Tendons have been largely evaluated using ultrasound-based techniques to detect mechanical and morphological changes [8, 14]. Among these techniques, ultrasound echo intensity has been used recently to provide quantitative analysis of the internal structure of the AT and PT. [13–17]

Ultrasound echo intensity analysis, also called gray scale analysis, is the evaluation of pixel-intensity distribution in a region of interest (ROI) within the ultrasound image that can be represented as a histogram of gray scale values [18]. Previous evaluation on the AT, [14, 15, 17] biceps tendon, and supraspinatus [19] has shown high reliability and validity. Moreover, tendon gray scale values have been used to discriminate between healthy and pathological tendons [14, 16].

Ski mountaineering is a winter sport that has gained popularity over the last few years. It was recently shortlisted for the winter Olympics, which caused an increase in the number of national and international skiing events. The Millet® Tour du Rutor extreme (Arvier, Aosta, Italy) is a 3-day ski mountaineering race competition consisting of 51.5 km, with 6900 m of an ascent. In ski mountaineering, athletes climb and descend mountains under their own power. During climbing, the athletes uses special skis equipped with adhesive skins that avoid backsliding. In the ascending phase, the AT is particularly loaded, given the high magnitude of ankle-knee motion and the repetitive pushing phase when the foot is in a tip-toe position.

In the descent phase a sustained tension of the quadriceps muscle-tendon unit is required to maintain and appropriately modify the knee angle during different trajectories [20]. Therefore different loading patterns are expected during the two phases.

Given the high load demand on the muscle-tendon unit of both the PT and the AT during ski mountaineering, and the acute effect of loading on morphological tendon parameters, the aim of this study was to evaluate if a prolonged ski mountaineering race could modify tendon properties measured with ultrasound echo intensity parameters.

## Methods

### Participants

Twenty well-trained athletes (seven women, 13 men) participating in the 19th Millet® Tour du Rutor extreme (Arvier, Aosta, Italy) were enrolled in the study.

Inclusion criteria: healthy ski mountaineering athletes, aged between 18 and 50 years.

Exclusion criteria: history of tendon injury and/or foot and knee surgery, or any painful episodes in the lower limbs within the previous year; history of connective tissue, metabolic, or endocrine diseases; systemic inflammatory disorder; rheumatoid arthritis, spondyloarthritis, hypercholesterolemia, and/or under steroid and/or estrogen medications at the time of the investigation. None of the participants screened were excluded.

The race consisted of three consecutive days of ski mountaineering over different racing tracks.

*Day-1* The “Arp-Vieille” stage consisted of a 19-km race with a 2639-m difference in altitude. *Day-2* The “Feleumaz” stage consisted of a 14-km race with a 1989-m difference in altitude. *Day-3* The “Rutor stage” consisted of an 18.5-km race with an elevation change of 2294 m. All athletes enrolled in the study completed the three-day race.

This study was approved by the Azienda USL Valle d’Aosta Ethics Committee (protocol no. 23/03/2018.0026243.I) and conducted according to the criteria set by the declaration of Helsinki. Each participant signed an informed consent form before participating in the study.

### Procedures

Self-reported demographic information (height, weight, age and leg dominance) were captured during the first measurement session. Leg dominance was determined by asking: “If you would shoot a ball on a target, which leg would you use to shoot the ball?”

The ultrasound assessment of the participants was performed one day before the start of the race and immediately after each of the three stages. Athletes were asked to report to the data collecting room as soon as possible after the end of each stage, and no more than 45 min elapsed between the end of the race and the start of data acquisition.

**Fig. 1:**
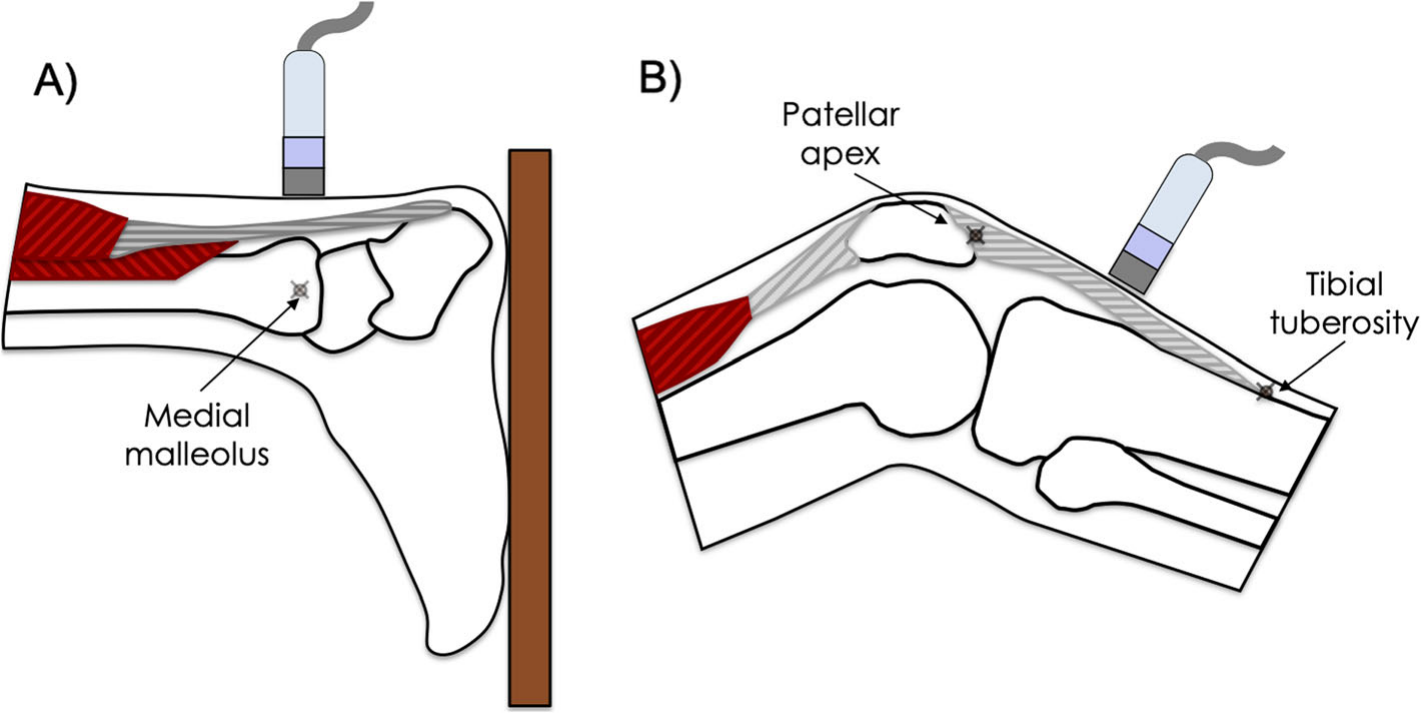
Schematics of the participant position and the position of the ultrasound probe. **a**) The Achilles tendon was analyzed at the level of the medial malleolus. **b**) The patellar tendon was analyzed half-way between the apex of the patella and the tibial tuberosity

The left and right AT and PT of each athlete were scanned in longitudinal and transverse planes. Participants were asked to lie in a prone position with feet in contact with a wall, to ensure the ankle was in a 0° flexion/extension. Longitudinal and transverse images of the AT were acquired at the level of the medial malleolus (mid-tendon) (Fig. 1a). Subsequently, participants were asked to lie in a supine position with the knee slightly flexed (at approximately 20°) over a round pillow. In this position, images of the PT were acquired in a transverse plane between the tibial tuberosity and the apex of the patella (Fig. 1b). Three images at each acquisition site were obtained, and thickness, CSA, and tendon echogenicity means were calculated.

### Ultrasound imaging system specifications

Ultrasound examination was performed using a portable ultrasound system (MyLab Alpha, Esaote, Genoa, Italy) equipped with a linear probe (SL1543) in the frequency range of 3/13 MHz. Time gain compensation was the same for all the depths, and global gain was set at 50% of the range. During the study, all system-setting parameters, as well as other options (e.g., persistence, dynamic range, and resolution), were the same for each participant. All data were acquired by a physical therapist with seven years of practice in ultrasound imaging and previous specific training in ultrasound imaging of tendons.

### Image analysis

Transverse ultrasound images were analyzed using custom MATLAB® software (MathWorks Inc., Natick, MA, USA). Morphological measurements (thickness and CSA) were calculated using standard calipers, whereas echogenicity was calculated using a semi-automatic (fixed-width) tracing procedure, as previously described [14, 17]. This procedure has been evaluated on the AT and its reliability has been tested in a previous study (ICC 0.94) [17]. With this semi-automatic procedure, the edges of the ROI within the tendon could be excluded, enabling proper evaluation of the tendon without considering the image artifacts due to the rounded shape of the lateral part of the tendon in the transverse scan.

**Fig. 2:**
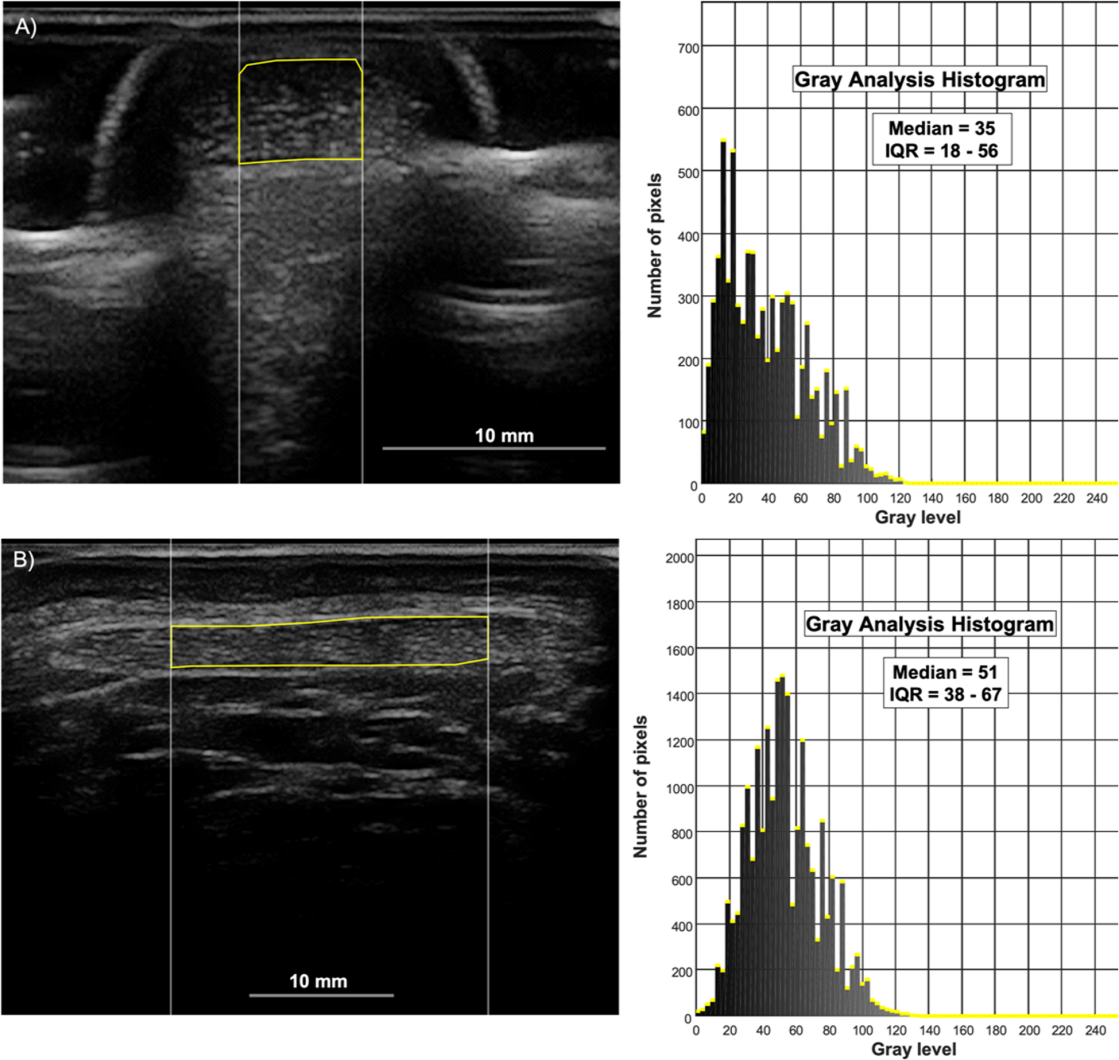
**a)** Transverse B-mode ultrasound image of the Achilles tendon with corresponding gray scale histogram. **b)** Transverse B-mode ultrasound image of the patellar tendon with corresponding gray scale histogram

The gray scale values of all pixels within the ROI are presented as a histogram (Fig. 2), from which the median and interquartile range (Q1-Q3) of the gray levels were extracted. Each pixel within the gray scale image ranged from 0 (black) to 255 (white), according to the depth of gray levels in a B-mode image. Echogenicity was represented by the median value of the gray scale distribution.

The gray scale values for the AT were calculated for the central 0.5 cm of the entire depth of the tendon CSA (Fig. 2a); whereas those for the PT were calculated for the central 2 cm (Fig. 2b). These values where chosen to exclude the ROI edges subjected to artifacts in all images of all participants.

### Statistical analysis

The Statistical Package for the Social Sciences (SPSS) version 24 (SPSS, Chicago, IL, USA) was used to perform statistical analysis. Descriptive statistics (Median; Q1-Q3) were used to characterize the entire sample. The Shapiro–Wilk test was used to evaluate normal distribution of the data. For non-parametric, related sample, the Wilcoxon signed-rank test was then used to assess whether there were significant differences in AT and PT thickness, CSA between left and right tendons, and between the dominant and non-dominant leg.

As the data were not normally distributed, the Friedman test with post hoc pairwise comparison was used to determine the differences in thickness, CSA, and echogenicity between the pre-race measurements and all of the three-day race for both dominant and non-dominant leg. Statistical significance was set at α = 0.05.

## Results

Twenty well-trained athletes with a mean age, height, and weight of 34 ± 10 years, 172.4 ± 9.0 cm, and 64 ± 10.4 kg, respectively were recruited, 14 were right leg and 6 left leg dominant.

Descriptive statistics of the values of the tendons in the dominant and non-dominant leg are presented in Table 1.

**Table 1:** Descriptive statistics for the Achilles and patellar tendon

	**Achilles tendon**	**Pre Race**	**After Race 1**	**After Race 2**	**After Race 3**
Dominant	Thickness (mm)	4.62 (4.37–4.85)	4.33 (3.80–4.55)	4.35 (3.98–4.67)	4.28 (3.95–4.78)
	CSA (mm^2^)	42.7 (40.3–44.2)	38.9 (35.4–40.7)	36.6 (35.2–38.9)	37.3 (35.2–39.9)
	Echogenicity	30.3 (28.3–32.5)	36.7 (31.7–40.3)	35.7 (30.9–40.1)	39.5 (36–42.9)
Non-Dominant	Thickness (mm)	4.98 (4.48–5.2)	4.04 (3.74–4.41)	4.31 (4.02–4.61)	4.21 (3.93–4.57)
	CSA (mm^2^)	43.6 (40.4–46.1)	39.2 (34.5–43.3)	39.9 (35.5–43.1)	39.5 (36.5–44.6)
	Echogenicity	30.5 (28.2–35.3)	37.4 (33.2–42.9)	38.2 (34.7–40)	42.9 (38.6–47.4)
	**Patellar tendon**	**Pre Race**	**After Race 1**	**After Race 2**	**After Race 3**
Dominant	Thickness (mm)	2.87 (2.51–3.13)	2.77 (2.59–3.25)	2.87 (2.45–3.38)	2.85 (2.46–3.35)
	CSA (mm^2^)	82.6 (74.4–96.3)	83.2 (70.6–105.9)	86 (74.5–106.4)	83.2 (70.2–97)
	Echogenicity	44.7 (35.5–58.1)	51 (44.4–61)	48 (41.3–62.5)	52.7 (45.9–65.8)
Non-Dominant	Thickness (mm)	2.84 (2.51–3.13)	2.99 (2.55–3.47)	2.90 (2.64–3.24)	2.95 (2.66–3.32)
	CSA (mm^2^)	83.2 (69.5–91)	80.2 (74.7–100)	82.5 (74.8–98.7)	82.3 (72.8–101)
	Echogenicity	49 (42.7–59.2)	55.5 (45.2–63.8)	54.9 (45.3–68.3)	57.5 (49.4–69.4)

Data are presented as median and IQR(Q1-Q3)

**Fig. 3:**
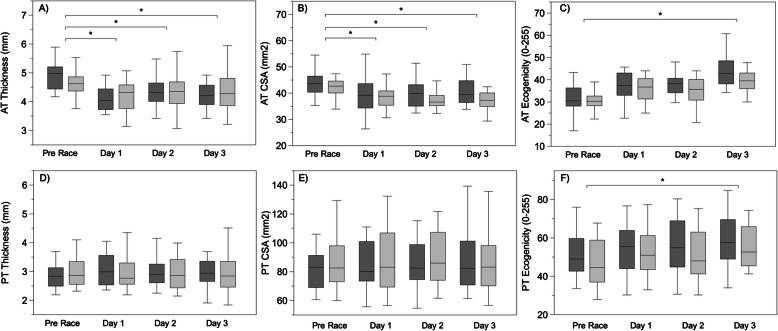
Achilles tendon thickness **a**), cross-sectional area **b)**, and echogenicity **c**) values for the non-dominant (dark gray) and dominant (light gray) leg, at the four evaluation times. Patellar tendon thickness **d**), cross-sectional area **e**), and echogenicity **f**) values for non-dominant (dark gray) and dominant (light gray) leg, at the four evaluation times. The box represents the median with the first and third quartile (Q1-Q3), whiskers represent the minimum and maximum value. * *p* < 0.05, indicates significant differences between the pre-race and post-race evaluation. (post-hoc analysis)

Wilcoxon signed-rank test showed significant differences in AT and PT thickness and CSA between the left and right tendon (*p* > 0.05) and between the dominant and non-dominant leg (*p* > 0.05). Therefore, a Friedman test for related samples was used to separately analyze the dominant and non-dominant leg. Post-hoc analysis revealed the following significant difference. The pre-race measurements and all of the three-day race evaluations for AT thickness and CSA, showing a decrease in tendon morphological parameters for both the dominant and non-dominant leg (*p* < 0.05). (Fig. 3).

Tendon gray scale values increased for both dominant and non-dominant leg between the pre-race measurement and the last race (*p* < 0.05). (Fig. 3). For the PT, a significant difference (*p* < 0.05) was observed for both the dominant and non-dominant leg only in the echogenicity between the pre-race measurement and the last race (*p* < 0.05). (Fig. 3).

## Discussion

To our knowledge, the present study is the first to report changes in morphological parameters based on ultrasound imaging before and after a prolonged ski mountaineering racing. Changes in tendon thickness, CSA, and echogenicity revealed an immediate response of the tendon to acute loading. These findings provide further evidence of the behavior of the tendon during loading, particularly, its ability to react immediately to a high-intensity training stimulus.

### Achilles tendon

Ski mountaineering is a high-impact sport that engages the entire musculoskeletal system. In particular, the AT, which is involved in the steep ascending phase, is put under tension from repetitive ankle-knee movements. This repetitive loading pattern could induce an acute response of the tendons, which seems to be attributed more to the load magnitude and contraction intensity than to the type of contraction [8]. The present study showed a significant reduction in tendon thickness and CSA between the pre-race measurement and each of the three post-race evaluations.

In a previous evaluation of AT thickness during eccentric and concentric exercises in healthy participants [11] and various in vitro studies, [21–23] cyclic loading has been proposed to exude water from the tendon with a consequent decrement in tendon dimensions. Whereas the water content present in the core of the tendon moves toward the peritendinous space [24, 25].

Our findings are in line with previous studies and tendon gray scale values are consistent with those of the morphological parameters, showing increased levels for the dominant, and for the non-dominant leg. (Table 1).

Tendon images showed a brighter internal pattern when subjected to a high-intensity load, compared with pre-race measurements. This brightness of the B-mode image could be related to the reduced water content within the tendon, as fluids in ultrasound images appear darker than connective tissues. This water movement can be attributed to the realignment and stretching of collagen fibrils during mechanical loading [11, 24]. The increased compressive force within the tendon and reduction of the interfibrillar space generate fluid movement outside of the tendon as a consequence of positive hydrostatic pressure.

Similarly, the present study showed a significant reduction in the CSA of the AT, confirming that repetitive loading can exude water from the tendon, and even the CSA is affected by this change in water content. Previous studies have reported an unchanged AT CSA after running or passive stretching [26, 27]; however, in the present study, the athletes were subjected to a prolonged race with a comparatively higher tendon stimulus. Moreover, in the recent systematic review of Bohm et al., [8] the authors suggest that material properties of tendons (i.e., stiffness and Young’s modulus) react earlier to loading compared to morphological properties (i.e., CSA). However, in that systematic review, the immediate effect of loading was not evaluated, and early tendon changes are considered to occur after 8–12 weeks of intervention.

### Patellar tendon

Tendon structure and properties differ across the body. Given the wide range of functional requirements during human movement, morphology and behavior can vary between tendons. The. PT is shorter and stiffer than the AT even if both are mainly designed for the storage and release of elastic energy [28].

Although some studies have reported a difference in the stiffness and CSA of the PT after resistance training, [6, 20] there is a paucity of literature evaluating the changes in morphological parameters induced by acute exercise.

The load applied to the quadriceps muscle-tendon unit, and more specifically, the PT, during downhill ski sports (i.e., alpine skiing and ski mountaineering) is mostly due to the necessity to maintain a flexed-knee position and modify the knee angle during different trajectories [20]. An acute response of the tendon tissue could be present, given the amount of strain generated during the downhill period, in which complete stress relief of the PT is not possible, owing to the absence of an unloading phase, as occurs in other more cyclic physical activities.

In the present study PT thickness and CSA were unchanged immediately after the races. Conversely, gray scale values of the PT showed a significant increase between the pre-race measurements and the end of the third race. The morphological properties of the PT were unchanged after the 3 days of racing, indicating a difference in its behavior in comparison to the AT.

Owing to the differences in function and type of loading during the activity, the stimulus induced by ski mountaineering was not sufficient to elicit changes in PT thickness and CSA. Hypertrophy of the PT following years of training in specific sports and in response to high-intensity, resistance training has been shown [6, 7]. In contrast, no change in the morphological properties of the PT has been reported after alpine skiing training of 12 weeks [20]. The differences between studies could be attributed to the behavior of the tendon, which undergoes changes only when a certain load threshold is overcome.

In the present study, the load applied to the tendon was probably not high enough to enable a morphological acute response in comparison to the high-intensity training proposed in previous studies [7, 20]. Moreover, hypertrophy of the PT seems to occur in a specific region of the tendon as opposed to its entire length. Hypertrophy has been observed, especially near the osteo-tendinous junction [7]. In the present study, the central part of the tendon was evaluated. Any possible change in the morphological properties of the PT away from the evaluation site could have been missed following this measurement protocol. However, we cannot rule out any PT response within the measurement error of the US imaging.

A significant difference was observed in the echogenicity of the PT. As reported for the AT measures, the gray scale levels were increased after each of the three races, showing a change in the internal pattern of the tendon. Similar to the AT, an increase in gray levels resulting in a “brighter” tendon is likely related to the movement of fluid from within the core of the tendon to the peritendinous space. A previous study, [13] using a slightly different methodology to evaluate the PT echogenicity, showed a similar increase in gray scale values after a squatting exercise session. Although this phenomenon in the PT are less described in the literature, changes in the internal pattern of the PT possibly occur prior to morphological changes. These early changes in material properties, without morphological changes, could be attributed to changes in the alignment of collagen fibers, loss of collagen crimping, [29] and the cellular response [30] due to loading.

Moreover, is important to remark that given the PT is less exposed to sliding and friction load compared to AT, the extent of peritendinous structure and the fluid present between the paratenon and the epitenon are probably reduced. The lack of morphological changes in the PT reported in this study could be related to a minor fluid movement in a tendon where peritendinous structures and fluid are less present. Differences in peritendon extent and size between the two tendons should be further investigated.

The presented study has some limitations that needs to be acknowledge. Reliability of the ultrasound echo intensity technique was evaluated only on AT; no data on PT are available. Ultrasound measurements were obtained at a standardized site in the mid-Achilles and PT; region-specific morphological changes could not have been confirmed by this study. Furthermore, baseline values were taken the day before the start of the three-day race. Due to logistical aspects, the recording of baseline measurements immediately before each of the three races was not possible. We could not have evaluated whether the morphological parameters returned to baseline values after rest; neither could we have excluded a cumulative effect of the different races. Lastly, there is a lack of control group; incidental walking has shown to be sufficient to induce Achilles tendon thickness changes [31] therefore a control group could have highlighted possible differences between high and low load activities.

The AT and PT of healthy athletes are highly responsive to an acute increase in mechanical load. An exhausting ski mountaineering race seems to reduce the thickness and CSA of the AT, and increase tendon echogenicity. Conversely, the PT showed increased tendon echogenicity without any notable change in morphological parameters. The differences in anatomy and function between the two tendons and the opposite loading patterns of ski mountaineering could explain the differences between their structures during acute loading.

Tendon fluid flow and tendon loading are known to be important to provide nutrition at the tendon and stimulate the remodeling [11, 32].

Given the importance of fluid movement in the tendon, it can be speculated that the ability to react to the mechanical load, showing immediate structural and morphological changes, could be the normal behavior of the tendon that protect the structure against the risk of overload.

The sample analyzed in this study had a very high level of training, it could be interesting to evaluate if tendons of persons with a lower level of training, subjected to an acute load, shows the same behavior.

If the importance of the fluid movement as a protective factor to limit tendon overload will be confirmed by future studies, an easy and low-cost technology able to early detect these changes could be helpful to personalize the training load of athletes during the season.

## Conclusions

Possible detection of early structural changes due to mechanical loading prior to changes in tendon morphology could be helpful in gaining a better understanding of tendon behavior and pathology. Therefore, the addition of the new ultrasound-based technique proposed in the present study to classical ultrasound morphological measurements could provide a more comprehensive approach to the evaluation of tendons during load application.

## Data Availability

All data generated during this study are included in this published article. Ultrasound images of the entire sample of the current study are available from the corresponding author on reasonable request.

## Abbreviations

CSA: Cross-sectional area
AT: Achilles tendon
PT: Patellar tendon
ROI: Region of interest
